# Whole-Genome Sequence of *Chlamydia gallinacea* Type Strain 08-1274/3

**DOI:** 10.1128/genomeA.00708-16

**Published:** 2016-07-21

**Authors:** Martin Hölzer, Karine Laroucau, Heather Huot Creasy, Sandra Ott, Fabien Vorimore, Patrik M. Bavoil, Manja Marz, Konrad Sachse

**Affiliations:** a RNA Bioinformatics and High-Throughput Analysis, Faculty of Mathematics and Computer Science, Friedrich-Schiller-Universität, Jena, Germany; b University Paris-Est, Agence Nationale de Securité Sanitaire de l’Alimentation, de l’Environnement et du Travail (ANSES), Animal Health Laboratory, Bacterial Zoonoses Unit, Maisons-Alfort, France; c Institute for Genome Sciences and Department of Microbiology and Immunology, University of Maryland School of Medicine, Baltimore, Maryland, USA; d Department Microbial Pathogenesis, University of Maryland School of Dentistry, Baltimore, Maryland, USA; e Leibniz Institute on Aging, Fritz Lipmann Institute, Jena, Germany; f Institute of Molecular Pathogenesis, Friedrich-Loeffler-Institut (Federal Research Institute for Animal Health), Jena, Germany

## Abstract

The recently introduced bacterial species *Chlamydia gallinacea* is known to occur in domestic poultry and other birds. Its potential as an avian pathogen and zoonotic agent is under investigation. The whole-genome sequence of its type strain, 08-1274/3, consists of a 1,059,583-bp chromosome with 914 protein-coding sequences (CDSs) and a plasmid (p1274) comprising 7,619 bp with 9 CDSs.

## GENOME ANNOUNCEMENT

*Chlamydia gallinacea* is an obligate intracellular bacterium classified under the single genus *Chlamydia* (1) within the family *Chlamydiaceae*. Comparative genetic analysis recently revealed its status as a separate species, with *C. avium* and *C. psittaci* being the closest relatives (2). Epidemiological observations suggest that the main host of *C. gallinacea* is domestic chicken (3–6), alongside turkey, duck, and guinea fowl. Its etiological importance is still subject to investigation. Experimentally infected chickens remained asymptomatic but showed reduced body-weight gains (6). Some observations suggest a zoonotic potential (3). Coinfections with *C. psittaci* seem to be common (7). Comparison of Chinese and European isolates have suggested high intraspecies diversity with 13 different *ompA* genotypes (6).

Whole-genome sequence studies will facilitate elucidation of unresolved issues. In a previous paper, we reported a partially assembled whole-genome sequence of *C. gallinacea* type strain 08-1274/3 (2) (NZ_AWUS00000000.1). Whole-genome sequencing was conducted at the Institute for Genome Sciences (University of Maryland, Baltimore, MD). Briefly, Illumina-sequenced reads of an average length of 250 nucleotides (nt) and genome coverage of 1,949× were assembled using CLC bio version 6.0.1, which resulted in four scaffolds sized 630,796 bp, 228,666 bp, 185,564 bp, and 7,088 bp (GenBank assembly accession GCA_000471025.1).

In the present work, the data set was subjected to *de novo* assembly. Nonchlamydial reads pertaining to host DNA (from culture in embryonated eggs) were identified through mapping to the *Gallus gallus* genome using Segemehl (8). The remaining reads were assembled using SPAdes version 3.7.0 (9) with *k*-mer values of 21, 33, 55, 77, 99, and 127, the -careful option, and automatic coverage cutoff, which yielded 22 contigs. Of those, 16 contigs were identified as sequencing or assembly artifacts. Two other contigs were assigned through BLAST to *Enterobacteria* phage phiX174 (positive control in DNA sequencing) and *Gallus gallus* mitochondria, respectively. Thus, the assembly resulted in scaffold 1 (643,147 nt), scaffold 2 (228,815 nt), and scaffold 3 (185,839 nt), all representing the chromosome, and scaffold 4 (7,619 nt) representing plasmid p1274. To close the gaps, primer sites were selected in flanking scaffold regions. The primers were used in PCR to generate DNA fragments of 600 to 800 bp (gap 1) and 1,300 to 1,500 bp (gap 2), which were sent to Eurofins Genomics (Ebersberg, Germany) for Sanger sequencing. Alignment of Sanger sequences to scaffolds 1 to 3 using BLAST and MAFFT (10) eventually enabled closure of the gaps.

The complete chromosomal sequence comprises 1,059,583 bp. Provisional annotations using Prokka (11) revealed 914 protein-encoding genes and 46 noncoding RNAs, including 39 tRNAs, three rRNAs, and one tmRNA. The size of plasmid p1274 was determined to be 7,619 bp with nine proteins encoded. The average G+C content of the genome is 37.9 mol%.

This is the first report of a completely assembled genome sequence of *C. gallinacea*. It can serve as a reference genome for future studies.

### Nucleotide sequence accession numbers.

The updated whole-genome sequence data of *C. gallinacea* type strain 08-1274/3 and its plasmid p1274 have been deposited in NCBI GenBank under accession numbers CP015840 and CP015841, respectively.
